# High throughput analysis of leaf chlorophyll content in sorghum using RGB, hyperspectral, and fluorescence imaging and sensor fusion

**DOI:** 10.1186/s13007-022-00892-0

**Published:** 2022-05-03

**Authors:** Huichun Zhang, Yufeng Ge, Xinyan Xie, Abbas Atefi, Nuwan K. Wijewardane, Suresh Thapa

**Affiliations:** 1 College of Mechanical and Electronic Engineering, Nanjing Forestry University, Nanjing, 210037 China; 2 Co-Innovation Center of Efficient Processing and Utilization of Forest Resources, Nanjing Forestry University, Nanjing, 210037 China; 3 Department of Biological Systems Engineering, University of Nebraska-Lincoln, Lincoln, NE 68583 USA; 4 Department of Computer Science and Engineering, University of Nebraska-Lincoln, Lincoln, NE 68583 USA; 5 Department of Agricultural and Biological Engineering, Mississippi State University, Starkville, MS USA

**Keywords:** Plant phenotyping, Chlorophyll content, Specific leaf weight, Partial least squares regression, High throughput, Image analysis

## Abstract

**Background:**

Leaf chlorophyll content plays an important role in indicating plant stresses and nutrient status. Traditional approaches for the quantification of chlorophyll content mainly include acetone ethanol extraction, spectrophotometry and high-performance liquid chromatography. Such destructive methods based on laboratory procedures are time consuming, expensive, and not suitable for high-throughput analysis. High throughput imaging techniques are now widely used for non-destructive analysis of plant phenotypic traits. In this study three imaging modules (RGB, hyperspectral, and fluorescence imaging) were, separately and in combination, used to estimate chlorophyll content of sorghum plants in a greenhouse environment. Color features, spectral indices, and chlorophyll fluorescence intensity were extracted from these three types of images, and multiple linear regression models and PLSR (partial least squares regression) models were built to predict leaf chlorophyll content (measured by a handheld leaf chlorophyll meter) from the image features.

**Results:**

The models with a single color feature from RGB images predicted chlorophyll content with R^2^ ranging from 0.67 to 0.88. The models using the three spectral indices extracted from hyperspectral images (Ration Vegetation Index, Normalized Difference Vegetation Index, and Modified Chlorophyll Absorption Ratio Index) predicted chlorophyll content with R^2^ ranging from 0.77 to 0.78. The model using the fluorescence intensity extracted from fluorescence images predicted chlorophyll content with R^2^ of 0.79. The PLSR model that involved all the image features extracted from the three different imaging modules exhibited the best performance for predicting chlorophyll content, with R^2^ of 0.90. It was also found that inclusion of SLW (Specific Leaf Weight) into the image-based models further improved the chlorophyll prediction accuracy.

**Conclusion:**

All three imaging modules (RGB, hyperspectral, and fluorescence) tested in our study alone could estimate chlorophyll content of sorghum plants reasonably well. Fusing image features from different imaging modules with PLSR modeling significantly improved the predictive performance. Image-based phenotyping could provide a rapid and non-destructive approach for estimating chlorophyll content in sorghum.

## Background

Chlorophyll constituents a major component of plant leaves and is a useful indicator of the overall health condition of the plant. Chlorophyll is the most important pigment for photosynthesis and growth. The determination of chlorophyll content in plant leaves can be used to investigate plant physiological and nutritional status, and consequently has important implications on crop stress and pests detection (such as in precision agriculture practices [23]). Generally, as plant stress levels increase, chlorophyll content tends to decrease [43]. Traditional approaches for the quantification of chlorophyll content mainly include acetone ethanol extraction, spectrophotometry and high-performance liquid chromatography. Such destructive methods based on laboratory procedures are time consuming, expensive, and not suitable for high-throughput phenotyping [15]. Using spectroscopy and a portable chlorophyll meter, several spectral indices have been identified, which can be used for predicting chlorophyll content in plant tissues in vivo [21]. However, manually operated portable chlorophyll meters are relatively biased, and spectroscopy techniques cannot measure the spatial distribution of chlorophyll in plant leaves [20]. Clearly these two methods are too labor-intensive to meet the needs of large-scale screening programs.

Advancements in phenotyping tools and methods for both proximal and remote sensing obtains massive amounts of plant imaging data. For chlorophyll content estimation, satellite imagery is useful for evaluation of moderate to large sized plots; whereas for smaller plots, remote sensing with unmanned aerial vehicles (UAVs) and proximal phenotyping are viable alternatives [19, 26, 35]. Satellite imaging could be especially valuable for replicated experimental trials at different locations to study genotype by environment interactions [6]. As trials are often performed with different stresses, proximal phenotyping could be a viable tool for evaluating chlorophyll estimation in greenhouse. For proximal phenotyping, sensors can be handheld or mounted on phenotyping platforms [3].

Rapid and non-invasive approaches for the screening and quantification of plant traits are particularly suitable for plant breeding programs. With the establishment of advanced technology facilities for high throughput plant phenotyping [10], estimating chlorophyll content of individual plants from their images becomes possible. This imaging based approach is more desirable than handheld chlorophyll meters, because it eliminates the involvement of human labor and further improves measurement throughput. Meanwhile, plants can be imaged by several imaging modalities, allowing accurate in vivo evaluation of many plant traits simultaneously. Conceivably, measurements of a large number of plants (e.g., hundreds) is possible, and measurements can be done at multiple time points across the entire life cycle of the plants which enables temporal dynamic analysis [46].

With the advancement in sensor technologies, high-throughput plant phenotyping (HTPP) has become more widely available to the research community, in particular the high-throughput imaging technology. Many researchers believe that image acquisition is no longer the challenge; the true bottleneck is in the analysis of thousands of plant images that are acquired in a short time window [29, 42]. Image-based features extracted from images, which contain the information on the morphological and biochemical traits of plants, enable effective use of genomic data to bridge the genotype-to-phenotype gap for crop improvement.

The imaging processing techniques have recently been used for remote detecting chlorophyll content. After obtaining the image, the chlorophyll content could be predicted by image segmentation and feature extraction. Image analysis has been used as an alternative for quantitatively predicting the chlorophyll distribution in some plants. In order to quantify the chlorophyll distribution in rice leaves, an integrated image analysis pipeline was developed for processing hyperspectral data. Three leaves were cut from the main stem of each rice plant and scanned using a hyperspectral camera. Models were built to quantify chlorophyll content and determined the important bands associated with it. The R^2^ values of the models were from 0. 827 to 0.928 [12]. An image analysis system was described for rapid determination of chlorophyll content of leaves of regenerated plants using the primary color components of red, green and blue. The leaves of micropropagated potato plants were scanned by the HP scan jet 3670 scanner to acquire images. A good correlations between the predicted and actual chlorophyll content was observed with RGB model and the R^2^ values were from 0.42 to 0.77 [48]. Researchers have also developed some algorithms to determine the correlation between chlorophyll content and color features. The leaves of four commercial cultivars were removed from the plants and placed flat on the light box and photographed by a digital color camera. The results showed that (R−B)/(R + B) is the most fitted function of RGB space to estimate the chlorophyll content of leaves [33]. In order to evaluate the correlation between photographic image-based hue parameters and classic, pigment extraction-based chlorophyll content determination, leaf disks with different pigment content at varying stages of naturally occurring senescence were cut form tobacco and grapevine leaves and photographed with a digital camera. The results suggested the relationship between photographic estimated and actual values of chlorophyll content was fitted well (R^2^ = 0.883) [34].

The previous research provided the parameter extraction method for one individual leaf image. The leaves were removed from the plant and placed flat and scanned by a camera, and then chlorophyll quantification model was constructed to analyze leaves' chlorophyll content. However, it is not possible to follow developmental stages of plants using such destructive methods. Additionally, the image analysis of an individual plant in vivo on a large scale has not been previously applied to the chlorophyll content estimation. It is possible to make spatially resolved predictions for the chlorophyll content at the whole plant level. It would be a tool to quantify the spatial distribution of chlorophyll content within the plant at multiple time points along the life cycle. If it can accurately work on predicting the chlorophyll content, the approach of image analysis could provide a rapid monitoring and screening technique to identify genotypes of high chlorophyll in large breeding populations for covering more growth stages.

The image fusion technique is employing by integrating complementary information from multi-image sensor data such that the new images are more suitable for the purpose of computer process. Effective combination of such sensors with different features could, therefore, extend the capabilities of the individual ones and provide a robust and complete description of an environment or process of interest, rather than using an individual source alone. Data fusion of image falls into three levels: data level, feature level, and decision level [11]. Although the concept of image fusion was proposed decades ago, the technology is still in its infancy in terms of increasing the reliability of proximal sensing systems for plant phenotyping, and is gaining popularity towards fast, high throughput and non-destructive evaluation of chlorophyll content.

In this paper, we present our work for non-destructive estimation of leaf chlorophyll content in sorghum using image-based traits derived from several imaging modules. We used a mini diversity panel of 15 sorghum genotypes exhibiting large variations in physical and physiological traits. The objectives of this study were to: (i) develop and validate an image analysis-based approach for non-destructive measurement of chlorophyll for individual plants, (ii) investigate how the chlorophyll estimation could be improved by including other auxiliary variables including DAS (day after sowing) and SLW (specific leaf weight), (iii) evaluate the potential use of the high throughput phenotypic images as a rapid tool to estimate spatial distribution of chlorophyll content within sorghum, and (iv) explore the fusion of multi-sensor images at the data level with different spatial and spectral resolutions in predicting plant chlorophyll content.

## Results

### Correlation of visible image with chlorophyll content

The linear model seems to be the best of those considered so far, justified by its common use in the literature. For our data, the linear method for estimating chlorophyll content (Chl) as a linear function of visible image was used. In these models the chlorophyll content (reading of MC 100 chlorophyll content meter) was the dependent variable, while DAS and the visible image were defined to be independent variables. For instance, the linear model is a function with the equation of Chl = a_0_ + a_1_R + a_2_DAS, where R is the specific leaf image of red value from visible image.

**Fig. 1:**
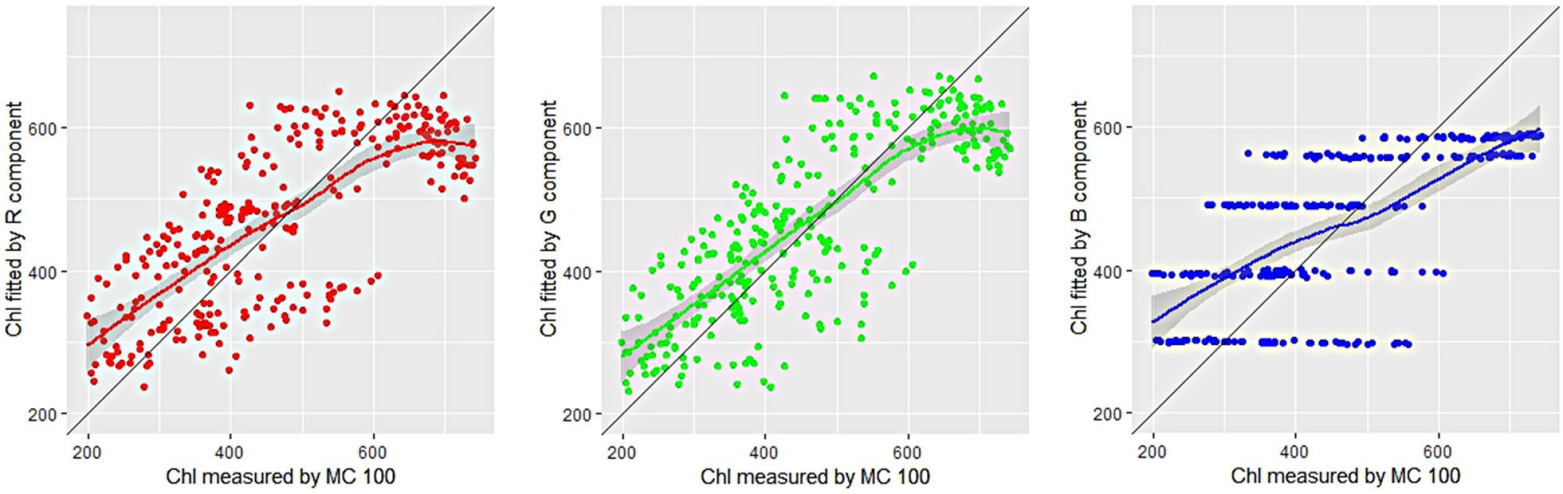
The correlations between the Chl values of measured by MC100 with predicted by R (red), G (green) and B (blue) component and DAS (day after sowing). *Chl* chlorophyll content, *MC100* portable leaf chlorophyll meter

The values of the three primary colors were linearly correlated to obtain the characteristic RGB models as described in Fig. 1. Among the values of three primary colors of the leaves of sorghum, R and G were negatively correlated with the chlorophyll content measured by chlorophyll content meter MC 100. It can be seen from Fig. 1 that in contrast to R and G, an increasing trend of mean brightness with chlorophyll content was observed with B color chromate. A good agreement between the value of G and chlorophyll content was observed, while value of R and B were poorly correlated with chlorophyll content which indicates the more relevance of G color than R and B for determining the chlorophyll content in living plants by using a single color component. The regression between chlorophyll data obtained from MC 100 values, R, G, B pixels and DAS showed a linear relationship (R^2^ = 0.56, 0.64 and 0.48).

**Fig. 2:**
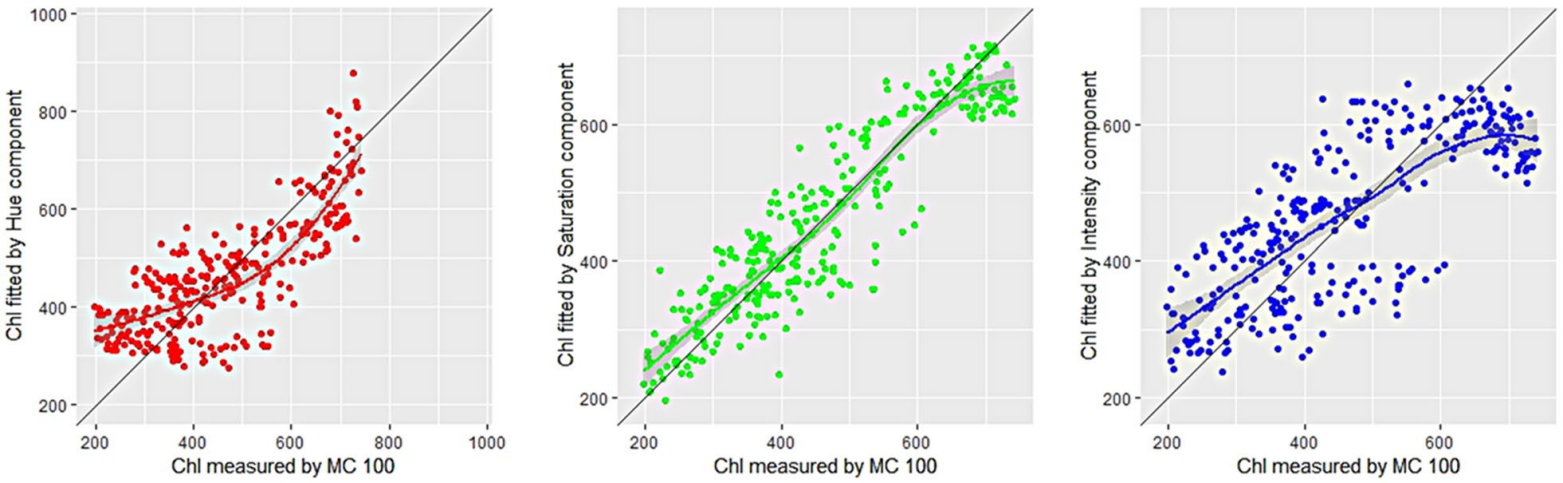
The correlations between the values of H (hue), S (saturation), I (intensity) and DAS (day after sowing) with chlorophyll content. *Chl* chlorophyll content, *MC100* portable leaf chlorophyll meter

From the visible image, primary colors R (red), G (green) and B (blue), was recorded. Spectral parameters such as H (hue), S (saturation) and I (intensity) were estimated from RGB values. The H, S, and I parameters were chosen from color space because it corresponds better to how people experience color than the RGB parameter set [31, 44]. Image information such as, hue, saturation and intensity color coordinates were also considered to study the relationship of color coordinates with chlorophyll content. Using the H, S and I components, the visible ‘‘greenness’’ of three leaves can be quantified and can be compared. A significant correlation was observed between the S parameter with chlorophyll content, while weaker correlation was observed with I parameter (Fig. 2). The R^2^ of hue effect on chlorophyll content was 0.61, which was supposed to distinguish the real colors on the leaves and be related to the chlorophyll content. However, it was worse than saturation, which was 0.85.

For Chl estimation in sorghum, Fig. 2 showed the correlation between hue, intensity and MC 100 reading (R^2^ = 0.61 and 0.57, respectively). Saturation gave a better Chl detection results with R^2^ = 0.85. HSI color model is found to achieve better fitting than RGB color model.

### Correlation of hyperspectral image with chlorophyll content

**Fig. 3:**
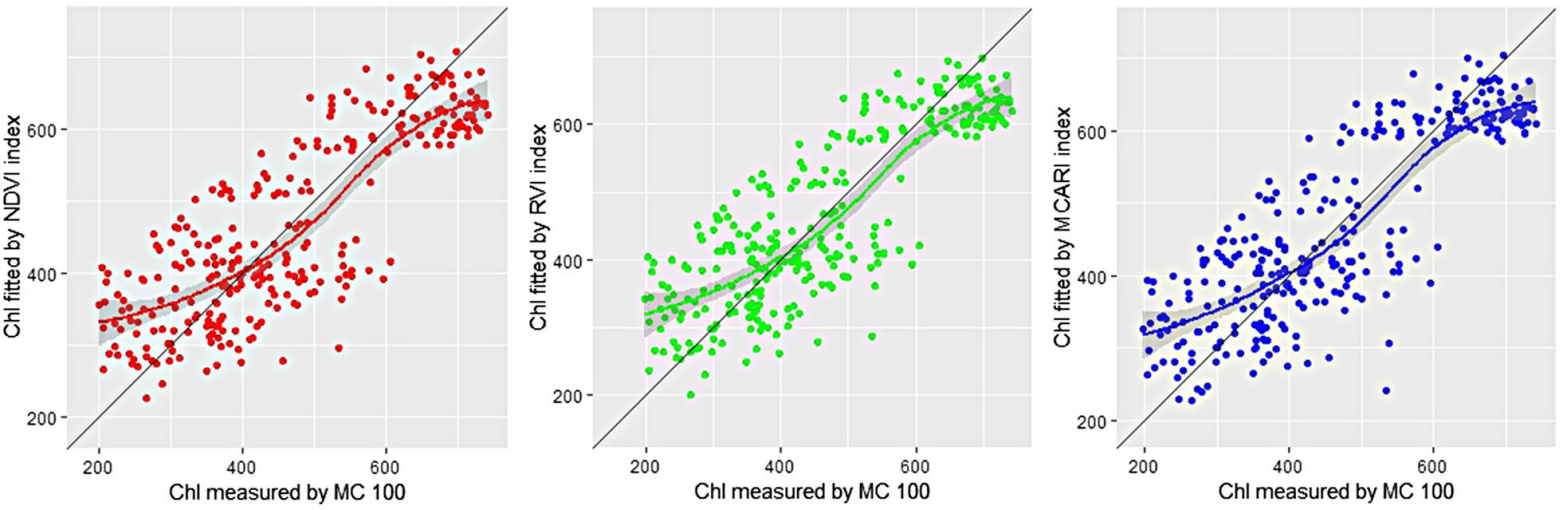
The correlations between the values of hyperspectral image with chlorophyll content. *Chl* chlorophyll content, *MC100* portable leaf chlorophyll meter

Figure 3 showed significant relationships between the Chl predicted by vegetation indices of hyperspectral images and MC 100 measured Chl. In fact, the obtained results are similar for the three indexes. On the other hand, the proposed method achieved better R^2^ to outperform liner regression method in predicting chlorophyll content.

Figure 3 showed the correlation between NDVI, RVI, MCARI and MC 100 reading (R^2^ = 0.68, 0.69 and 0.69, respectively). RVI and MCARI gave a slightly better Chl detection results with R^2^ = 0.69. It can be seen that normalized spectral index, ratio spectral index, and multi-band spectral index can predict the chlorophyll content well.

### Correlation of fluorescence image with chlorophyll content

**Fig. 4:**
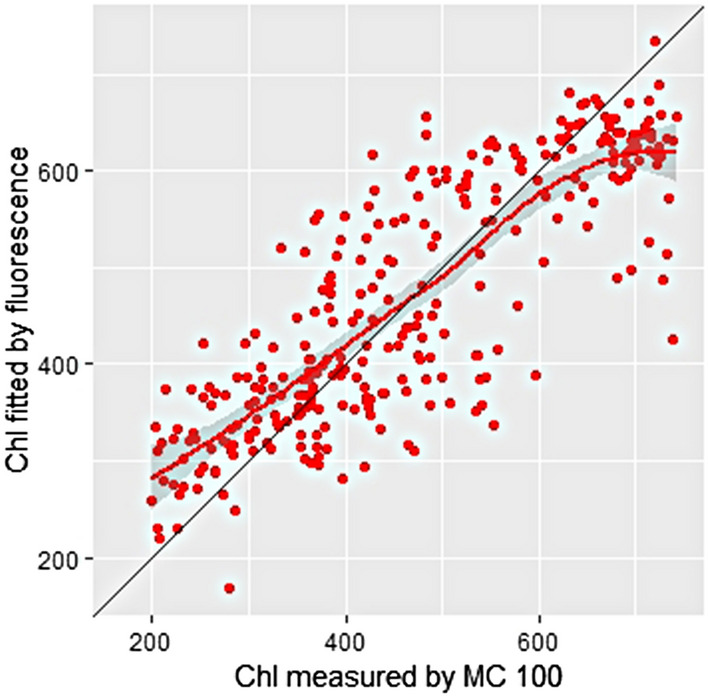
The correlations between the values of Fluo (fluorescence) image and DAS (day after sowing) with chlorophyll content. *Chl* chlorophyll content, *MC100* portable leaf chlorophyll meter

Data were subjected to simple regression analysis, with value from MC 100 chlorophyll meter as the dependent variable and greenness chlorophyll content form fluorescence image as the independent variable. The result was shown in Fig. 4 and R^2^ was 0.69. By non-destructively detecting the chlorophyll content and chlorophyll distribution of sorghum, it could provide a support for greenhouse crop growth evaluation and precision management.

### Correlation of different images with chlorophyll content

PLSR was developed by using the selected vegetation indices computed from different imaging data to model and predict chlorophyll content for sorghum plants. Hue, saturation, intensity, R, G, B, fluorescence, NDVI, RVI, MCARI, are first used, and then DAS (day after sowing) was added. Firstly, the models without DAS information are considered. The component number of 9 suggests the best result with all variables. To get the necessity for all variables included, the importance of all vegetation indexes is also examined. It shows that all vegetation indexes were important in constructing the model. Then the models with DAS information were considered. The component number of 8 guarantees the best result with all variables considered. Based on the absolute value for coefficients for each variable, the importance for each vegetation index is sorted. The larger the absolute value of the coefficient is, the more indispensable the corresponding variable is. Therefore, it indicated that the hue information is not necessary for constructing the PLSR regression with the component number of 9. The statistics to evaluations for each model are listed in Table 1.

**Table1:** Test results of using the selected vegetation indices computed from different imaging data to predict chlorophyll content for sorghum plants

			Calibration dataset with fivefold validation	Calibration dataset	Validation dataset	Entire dataset
Model 1 (without DAS)	Without feature reduction	R^2^	0.84	0.86	0.90	0.87
		RMSE	60.70	57.53	51.34	55.39
		RPD	2.49	2.62	3.16	2.77
Model 2 (with DAS)	Without feature reduction	R^2^	0.87	0.88	0.92	0.89
		RMSE	54.68	51.89	45.54	49.89
		RPD	2.76	2.90	3.57	3.08
	With feature ‘Hue’ reduction	R^2^	0.87	0.88	0.92	0.90
		RMSE	54.60	51.61	45.37	49.64
		RPD	2.76	2.92	3.58	3.09

Table 1 showed that PLSR regression models had R^2^ greater than 0.84 and RPD larger than 2.49, which suggested that PLSR regression models provided an accurate way to predict chlorophyll's content.

**Fig. 5:**
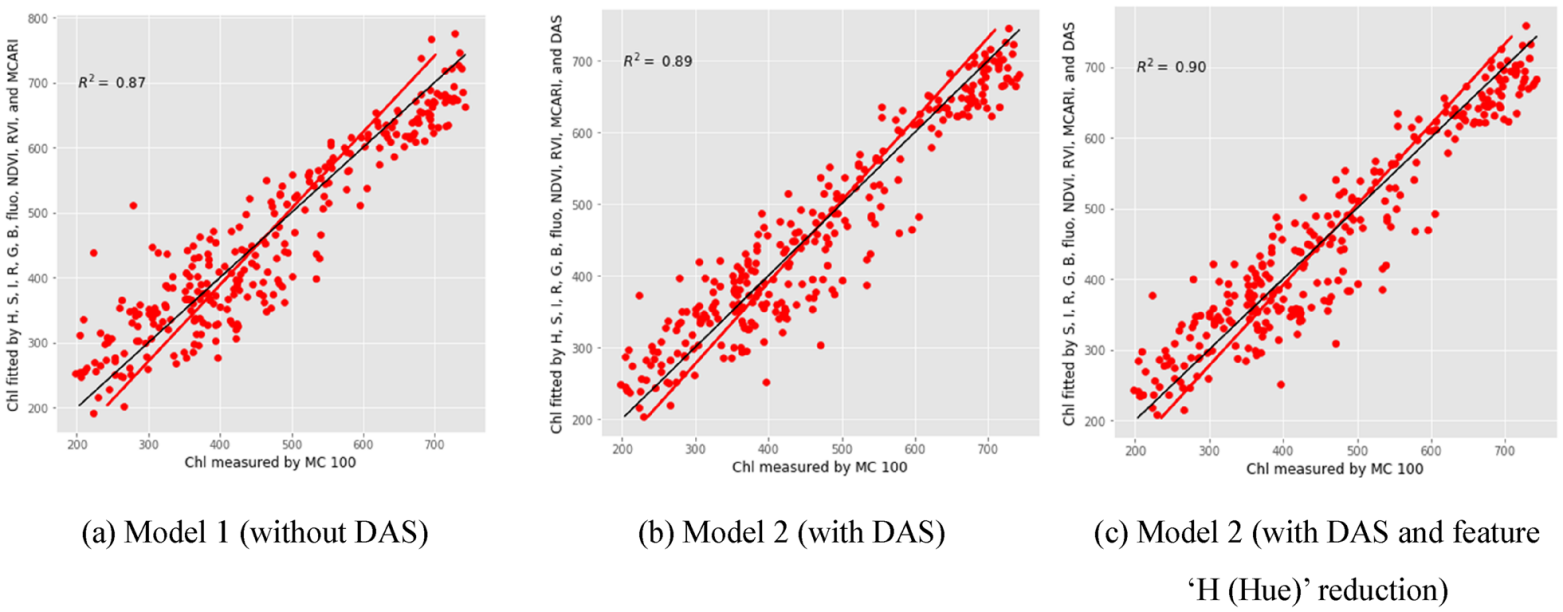
The correlations between the selected vegetation indices computed from different imaging data with chlorophyll content. *Chl* chlorophyll content, *MC100* portable leaf chlorophyll meter

It can be seen form Fig. 5 that the PLSR Model 2 (with DAS and feature ‘Hue’ reduction) has improved the R^2^ compared with the PLSR Model 1 (without DAS) and PLSR Model 2 (with DAS). Comparing the performance among different models, it can be concluded that (1) When more useful features are considered, the performance of the model becomes better. (2) Reducing unnecessary features in the model, the performance of the model becomes better.

## Discussion

In order to reduce the bias, we put forward a predictive model based on the specific leaf weight (SLW). SLW and single-leaf apparent photosynthesis (AP) have been shown to be positively correlated in field studies [4]. Sampling for SLW as a predictor of AP is not widely employed because it takes much time and energy and thus is not practical for evaluating large populations [45]. SLW is defined as the leaf dry weight per one-side area and it is sensitive to plant nitrogen status, light climate and several other stresses [13], so it is a key variable involved with or related to physiological processes occurring in the functioning of canopies. The observations across the images showed that chlorophyll content can be estimated as a multiple linear function of color component and SLW (Table 2). Therefore, chlorophyll content could be written as a linear form of images, DAS and SLW, i.e. Chl = b_0_ + b_1_R + b_2_DAS + b_3_SLW.

**Table 2:** Estimation error for different multiple linear models used to estimate chlorophyll content for various imaging technology

Imaging technology	Component	Regression	R^2^	AIC
Visible image	R	y = a_0_ + a_1_R + a_2_DAS	0.56	3630
		y = b_0_ + b_1_R + b_2_DAS + b_3_SLW	0.77	3440
	G	y = a_0_ + a_1_G + a_2_DAS	0.64	3569
		y = b_0_ + b_1_G + b_2_DAS + b_3_SLW	0.79	3413
	B	y = a_0_ + a_1_B + a_2_DAS	0.48	3680
		y = b_0_ + b_1_B + b_2_DAS + b_3_SLW	0.70	3513
	H	y = a_0_ + a_1_H + a_2_DAS	0.61	3589
		y = b_0_ + b_1_H + b_2_DAS + b_3_SLW	0.67	3539
	S	y = a_0_ + a_1_S + a_2_DAS	0.85	3309
		y = b_0_ + b_1_S + b_2_DAS + b_3_SLW	0.88	3252
	I	y = a_0_ + a_1_I + a_2_DAS	0.57	3621
		y = b_0_ + b_1_I + b_2_DAS + b_3_SLW	0.77	3440
Hyperspectral image	NDVI	y = a_0_ + a_1_NDVI + a_2_DAS	0.68	3488
		y = b_0_ + b_1_NDVI + b_2_DAS + b_3_SLW	0.77	3395
	RVI	y = a_0_ + a_1_RVI + a_2_DAS	0.69	3475
		y = b_0_ + b_1_RVI + b_2_DAS + b_3_SLW	0.77	3383
	MCARI	y = a_0_ + a_1_MCARI + a_2_DAS	0.69	3471
		y = b_0_ + b_1_MCARI + b_2_DAS + b_3_SLW	0.78	3375
Fluorescence image		y = a_0_ + a_1_Fluo + a_2_DAS	0.69	3519
		y = b_0_ + b_1_Fluo + b_2_DAS + b_3_SLW	0.79	3404

As the multiple linear model based on DAS and SLW proved to be better than the multiple linear models we considered, we compared our proposed model with the linear model described in Table 2. Multiple linear model based on DAS and SLW produced significantly larger R^2^, so the correlation of regression model introduced SLW has significantly improved. Similarly, low values of AIC also confirmed the model tested on DAS and SLW can satisfactorily estimate chlorophyll content. It can be seen from Table 2 that the maximum correlation coefficient of R^2^ = 0.88 was obtained with multiple linear regression based on DAS, SLW, and S (Saturation). The multiple linear regression based on DAS of S revealed the correlation coefficient of R^2^ = 0.85. The analysis also substantiated the potential use of H, S and I than the primary colors R, G and B. For hyperspectral index, NDVI, RVI, MCARI all shows that the adjustment of phenotypic image values for SLW greatly increases the accuracy of the prediction. The higher R^2^ values and lower AIC values confirmed the best fitted model after introducing parameter SLW that estimates the chlorophyll content (Table 2). Multiple linear regression model with MC 100 value as the dependent variable, and greenness chlorophyll content form fluorescence image and SLW as the independent variables gave the good estimation of chlorophyll content in leaves of sorghum. This analysis was to test hypothesis that specific leaf weight (SLW) could be one factor determining leaf chlorophyll content under different water and nutrition conditions. The influence of SLW on chlorophyll content and an improved simple method to determine chlorophyll content of sorghum by MC 100 chlorophyll meter was seen from Table 2.

**Fig. 6:**
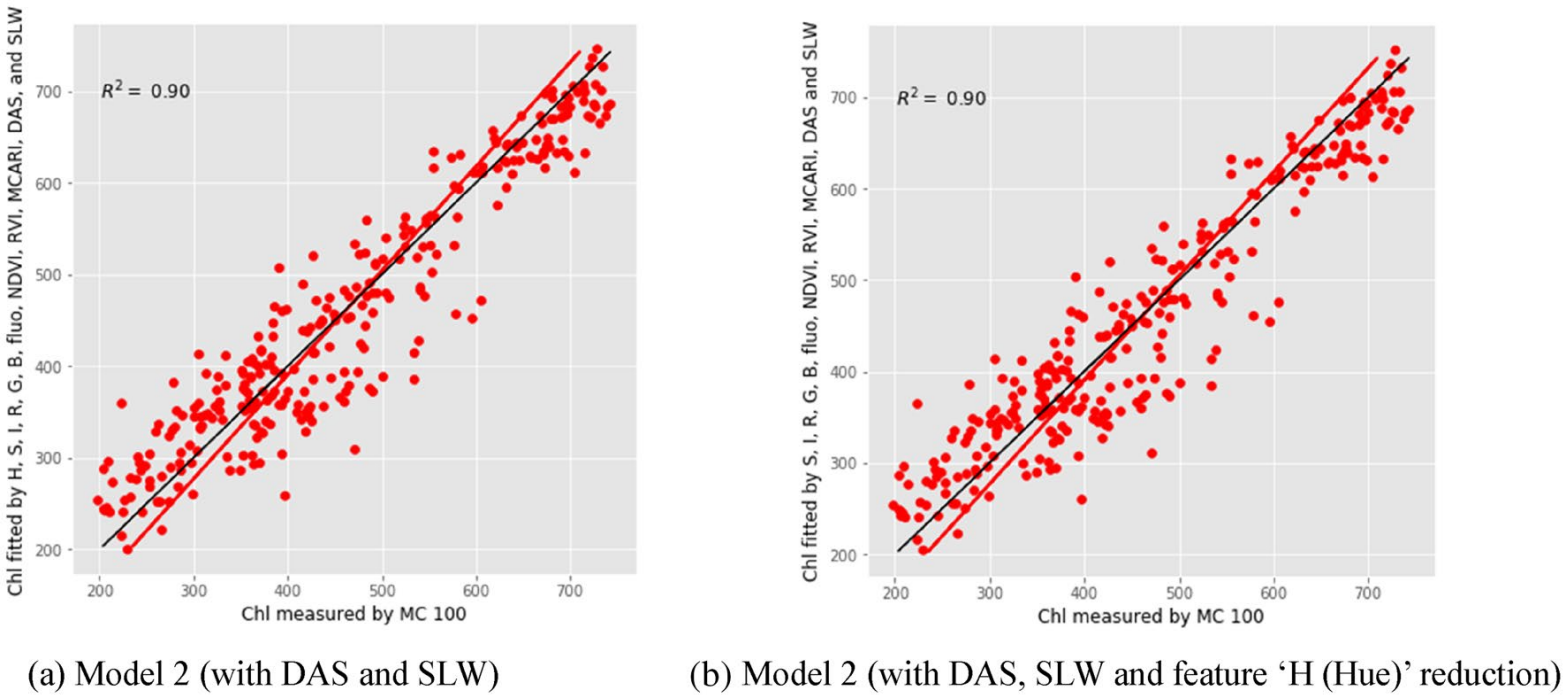
The correlations between the selected vegetation indices computed from different imaging data and SLW with chlorophyll content. *Chl* chlorophyll content, *MC100* portable leaf chlorophyll meter

Chl could be estimated quite satisfactorily with the selected vegetation indices computed from different imaging data for sorghum plants (Table 3). It can be seen R^2^ range from 0.87 to 0.92; whereas RPD is larger than 2.77. Therefore, the linear combination of saturation, intensity, R, G, B, fluorescence, NDVI, RVI, MCARI, DAS, and SLW, suggests the best regression model, as shown in Fig. 6. The red dots represent all samples, and the blue line is the prediction line from the model. It can be seen form Table 3 that the PLSR Model 3 (after adding DAS) has improved the R^2^ and RPD.

**Table 3:** Test results of using the selected vegetation indices computed from different imaging data and SLW to predict chlorophyll content for sorghum plants

			Calibration dataset with fivefold validation	Calibration dataset	Validation dataset	Entire dataset
Model 3 (with DAS and SLW)	Without feature reduction	R^2^	0.87	0.88	0.92	0.90
		RMSE	54.40	51.56	45.61	49.59
		RPD	2.77	2.93	3.56	3.09
	With feature ‘Hue’ reduction	R^2^	0.87	0.88	0.92	0.90
		RMSE	54.29	51.46	45.41	49.49
		RPD	2.78	2.93	3.58	3.10

Overall, these results confirmed the idea that the SLW, which was used as an additional input for predicting chlorophyll content in high throughput phenotypic image, plays a vital role in reducing the error. This can be seen in Tables 2 and 3 that R^2^ of the model was increased by including SLW.

As can be seen from Table 2, the values of error function used to minimize the differences between the experimental and predicted data, all the models based on DAS and SLW exhibited high R^2^ value and low values of AIC, thereby confirming better performance for models tested. This difference between the linear regression and multiple linear regression might have been due to introducing variable SLW, one of indicators for leaf thickness. The influence of leaf thickness on regression model contributed to better estimation of chlorophyll content by the chlorophyll meter. Leaf thickness changes according to leaf age and growth environment [18, 28]. Also, it has been demonstrated that reflectance increases and transmittance decrease with an increase in leaf thickness [49]. Thus, it is hypothesized that leaf thickness is one of the factors that determines chlorophyll content under different conditions (water conditions, and nutrition treatment).

Increasing SLW may improve leaf apparent photosynthesis. Pettigrew reported that plants grown under dryland production had a 12% increase in SLW, and he speculated that these leaves may have been denser or thicker than leaves of irrigated plants [40]. In response to drought, water-stressed plants had 12% more chlorophyll than well watered plants [7]. Campbell et al. analyzed the relationship between the SPAD-501 (SPAD) meter and total extracted chlorophyll (TChl) for leaf sets grown under greenhouse and field conditions, and found big difference. It has been suggested that the disparity in the models between experiments may partly be due to differences in leaf thickness. Field-grown leaves are typically thicker than greenhouse-grown leaves, and this is supported by the higher SLW values for the field-grown leaves [5]. The estimation of Chl with the SPAD over time may be confounded by changes in SLW. Peng et al. also demonstrated that thick leaves increased SPAD readings and thicker leaves (i.e. higher SLW) absorbed red light more than infrared light in leaves with similar chlorophyll content on the basis of leaf area [39]. SLW is in general an indicator of leaf thickness and the degree of mesophyll development within a leaf blade. The extent of mesophyll development largely determines the photosynthetic capacity of a leaf. Thus, SLW can potentially be used as an indirect measure of the photosynthetic characteristics of a leaf [25].

The possibility exists for why there was higher chlorophyll content in stressed plants: drought-stressed plants had smaller and thicker leaves, causing higher chlorophyll content. Well-watered plants may have translocated nutrient resources to new growing areas due to the fact that irrigated plants had an extended growing season resulting in lower chlorophyll readings [8]. The SLW data revealed stressed plants had thicker and denser leaves, which may have led to more chlorophyll per leaf and consequently SLW is an important contributing variable for predicting chlorophyll.

The objective of the present study is to develop a generalized method to estimate the chlorophyll content of sorghum from its high throughput phenotypic image. We have developed a method that significantly reduces the bias in chlorophyll content estimation of stressed plants. We have demonstrated that models that uses mixed variables of plant image’s greenness and SLW achieves this reduction and therefore the method we proposed can be used to compute more accurately the chlorophyll content of sorghum regardless of whether or not they are water and nutrition stressed.

As easy method for determining the chlorophyll content is using portable chlorophyll meter. Even in vivo chlorophyll determination can be made using SPAD-502 m that makes nondestructive and rapid measurements of leaf chlorophyll based on spectral transmittance properties of leaves [32]. However, chlorophyll meter provides that data only in arbitrary units rather than the actually amounts of chlorophyll per unit of leaf tissue.

In order to match the results of image processing to the value obtained by reference methods on Leaves 2, 3 and 4, empirically determined thresholds were evaluated on the percentage of the height of the plant. Segmentation using a fixed threshold was effective when the experiment was carried out in the greenhouse, because plant images were taken in imaging chambers with consistent lighting and background. Our image processing method focused on the plants in controlled environment, and we were trying to make image processing as straightforward as possible so as to match the results to the values from MC 100 in specific leaves more accurately. According to the results, the greenhouse based method was proven useful to quantify certain sorghum traits such as chlorophyll, but it may be of limited relevance and not directly translatable for field-grown plants.

Recently the image processing techniques have been used for remote sensing studies concerning plant monitoring projects. Optical sensors and sensing provide an unprecedented way to measure plant phenotypic parameters noninvasively to identify traits precisely. However, reports on the in-vivo analysis of chlorophyll content from high throughput phenotyping facility cannot be found in the literature. The use of the imaging techniques for in vivo characterization of leaf chlorophyll content at the plant level would provide information about the usefulness of the technology in non-destructive phenotyping, stress detecting, ranking, and selection of plants.

It seems that high throughput phenotypic image provides a simple, rapid, and nondestructive method to estimate the leaf chlorophyll content, and could be reliably exploited to predict the exact stress in sorghum. The present work demonstrated the potential for real time estimation of chlorophyll content by high throughput image analysis and DAS.

SLW is calculated as the ratio of leaf dry weight to fresh leaf area. Although there was not an instrument to directly and accurately measure leaf thickness in this study, the measurement of leaf thickness could be done nondestructively and relatively easily (compared to the manual measurement of SLW). As a result of technical advances, particularly the optical sensor and image processing, more methods of instrumental analysis without the destructive effect on the leaf samples have been reported [1, 24]. Therefore, it is reasonable to anticipate that a method or device for directly measuring leaf thickness could be developed and incorporated into the image analysis to provide chlorophyll content more accurately.

## Conclusions

In this study, a robust and accurate method has been developed for rapid and noninvasive determination of the chlorophyll content of sorghum leaves using visible, hyperspectral and fluorescence based image analysis. The results suggested that the three imaging systems combined with data fusion strategy, could be used synergistically to improve plant chlorophyll content prediction. The correlation was improved by the spectral properties along with two other parameters: DAS and SLW. It can be seen that adjustment of phenotypic image values for SLW increases the accuracy of the prediction. An image analysis method based on SLW may be an alternative choice for the real time prediction of chlorophyll content of plants. The potential of the imaging system in predicting chlorophyll has been discussed. It is concluded that imaging techniques can be a powerful tool for low-cost, nondestructive and high-throughput analysis of chlorophyll content.

Development of phenotyping tools and image processing methods for proximal sensing is of paramount importance in furthering the understanding of “phenomes” and the underlying genetics underpinning them for controlled growth conditions. We suggest that future work include the following aspects. Firstly, more advanced methods should be development and tested in plant segmentation and individual leaf identification. In our study, to match the results of image processing to the value obtained by reference methods on Leaves 2, 3 and 4 of a plant, empirically determined thresholds were evaluated on the percentage of the height of the plant. More general methods, especially those based on convolutional neural network and deep learning, could be leveraged to identify individual leaves more accurately. Secondly, this study tested the performance of data fusion strategy at the data level, and it was the first step in demonstrating the feasibility of the fusion of visible (RGB), hyperspectral, and fluorescence imaging systems with complementary spectral ranges for detecting chlorophyll content. It will be an interesting task to explore chlorophyll content estimation by fusing multi-sensor images at feature level and decision level. This would make different high throughput phenotyping imaging techniques more useful for chlorophyll content estimation research.

## Materials and methods

###  Plant material and growth conditions

The experiment was conducted at the Greenhouse Innovation Center of the University of Nebraska-Lincoln starting January 2019. Data collection occurred in April 2019. Fifteen different sorghum genotypes (each with 20 individual plants, 300 plants total) were used in this study, and the goal was to create a large variation in plant leaf property to validate HTPP image-based measurement.

**Fig. 7:**
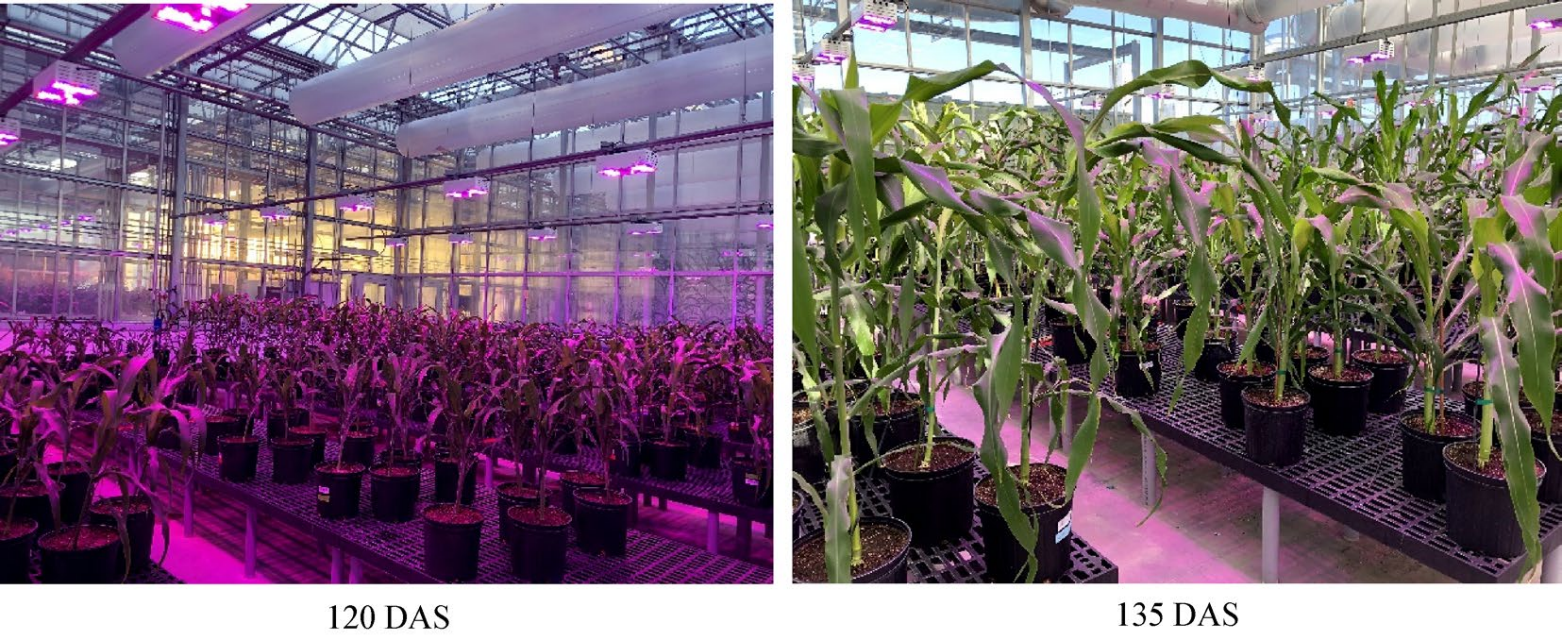
Photos of the planted sorghum with different DAS (days after sowing)

Some detailed greenhouse parameters are listed as follows. The temperature in the greenhouse was regulated between 25 and 27 °C during daytime and 20–22 °C during the night time. Relative humidity was maintained at ∼60%. The daily light intensity resulting from natural sunlight and the supplemental LED peaked at ∼350 μmol/m^2^/s photosynthetically active radiation. The supplemental LED had a photoperiod set to 12 h. The pots used were 25.72 cm in diameter and 23.18 cm in height, with a capacity of 8.52 L. The pot substrate was made by mixing Fafard germination soil and water. The visual differences between DAS were pronounced (Fig. 7).

The experiment was designed as a two-factorial design for each genotype (water regime and nutrition regime), with replicated five times for each combination. For the 20 plants in each genotype, 5 plants were randomly selected and assigned to one of the four treatment combinations: drought (D) and high nutrition condition (HN), drought (D) and low nutrition condition (LN), well-watered (HW) and high nutrition condition (HN), well-watered (HW) and low nutrition condition (LN). The estimated volumetric water content of soil for the drought group (maintain 5230 g of pot weight by adding water daily) was 30% and for the well-watered group was approximately 70% of field capacity. During the growth, the plants were watered every day to the targeted soil water content. For the nutrient regime, two different nutrient solutions (high-nutrition vs. low-nutrition treatments) were applied twice a week, and the detailed information of the solutions were as shown in Table 4.

**Table 4:** Composition of the nutrient solution to provide different levels of nutrition

Chemical	High nutrition g/L	Low nutrition g/L	Concentration ml/L
KNO_3_	82.15	8.21	8
Ca(NO_3_)_2_·2H_2_O	118.07	11.81	
KCl	0	46.13	
CaCl_2_·2H_2_O	0	66.16	
NH_4_H_2_PO_4_	14.38	1.44	8
MgSO_4_·7H_2_O	61.62	61.62	
KH_2_PO_4_	0	15.31	
H_3_BO_3_	0.28	0.28	1
MnCl_2_·4H_2_O	0.10	0.10	
ZnSO_4_·7H_2_O	0.06	0.06	
(NH_4_)_6_Mo_7_O_24_·4H_2_O	0.12	0.12	
CuSO_4_·5H_2_O	0.05	0.05	
Fe-EDTA	26.21	26.21	1

### High throughput imaging collection

High throughput images were collected on the sorghums prior to destructive sampling of plant leaf tissues. The sampled leaves were under stresses that affects chlorophyll content. This greenhouse was equipped with a high-throughput plant phenotyping system (Scanalyzer3D, LemnaTec GmbH, Aachen, Germany) [14]. The study collected visible (RGB), hyperspectral, and fluorescence images by using three imaging chambers. The imaging modules and their main parameters in these chambers are shown in Table 5. The hyperspectral imaging chamber is illuminated by two banks of halogen lamps (35 W, color temperature 2600 K), located on the ceiling above the plant and the other on the wall behind the imaging system. The chambers are designed to permit the imaging of plants up to a nominal height of 2.5 m. During test, the intact plants were loaded onto the conveyer belt and transported into the chambers for imaging.

**Table5:** Main parameters of the LemnaTec3D Scanalyzer imaging system

Imaging chamber	Camera maker	Position	Key parameters
Visible (RGB)	Basler	Side, Top	Resolution: 2454–2056 pixels 24 bit Spectral range: 400–700 nm
Steady state fluorescence	Basler	Side, Top	Excitation wavelength: 400–500 nm Measured emission wavelength:500–750 nm Resolution: 1038–1390 pixels 24 bit
Hyperspectral	Headwall	Side	Wavelength range: 550–1750 nm Spectral bandwidth: 5 nm number of bands: 243 Spatial resolution: 320 pixel line width Image formation: vertical scanning

### Sampling plant leaves for determination of phenotypic traits

Sorghum development was monitored throughout the growing cycle. Plant sampling was conducted when roughly 80% of the sorghum plants were undergoing flowering. The measurements were performed only on the main tiller. After image acquisition, the plants were destructively measured for the determination of phenotypic traits as described below.

From each plant, the 3 leaf samples were chosen except for the flag leaf (which is leaf 1). Leaf 2, 3 and 4 from the plant were cut at the stem and immediately weighed for fresh weight. Leaf Area (LA) of leaf 2, 3 and 4 was determined with a leaf area meter (LI-3100C, LI-COR Biosciences, Lincoln, USA). Chlorophyll content was estimated nondestructively with a portable chlorophyll content meter (MC-100, Apogee Instruments, Inc., Logan, UT). It was calibrated to measure chlorophyll content in leaves using the sensor’s build-in sorghum calibration with the unit of chlorophyll content being µmol/m^2^. The MC 100 value has already been found to provide the most accurate estimation of chlorophyll content in good correlation with leaf chlorophyll content extracted through organic solvent method [37]. Three sampling areas of approximately 64 mm^2^ (circle with 9 mm diameter) were taken from the same leaf for the determination of chlorophyll. That is, every leaf was estimated at the tip, middle and base sections to account for in-leaf variability, and the average of the nine spot measurements was regarded as the plant’s chlorophyll content value from that plant. The harvested plant leaves were then placed in a walk-in oven at 50 °C for 72 h, followed by the measurement of dry weight.

### Phenotyping image processing and data analysis

Image processing of the RGB, hyperspectral, and fluorescence images was done by using Matlab R2017a (MATLAB and Image Processing and Computer Vision Toolbox Release 2017a, The MathWorks, Inc., Natick, Massachusetts, United States). The major task of image processing was to extract plant pixels from RGB, hyperspectral, and fluorescence images from which image-based plant phenotypes can be derived.

As stated earlier, chlorophyll content was measured at Leaves 2, 3 and 4 of each plant. However, the cameras captured images of the entire plant including all leaves and the stem. In order to match what the chlorophyll meter measure to the images, efforts were made to confine the analysis on leaves 2, 3 and 4 of each plant image only and calculate their projected area. In the present study, the specific leaf image is defined as the leaf image amount (for example, greenness of image) per total projected area(pixel number).

#### Visible imaging acquisition

**Fig. 8:**
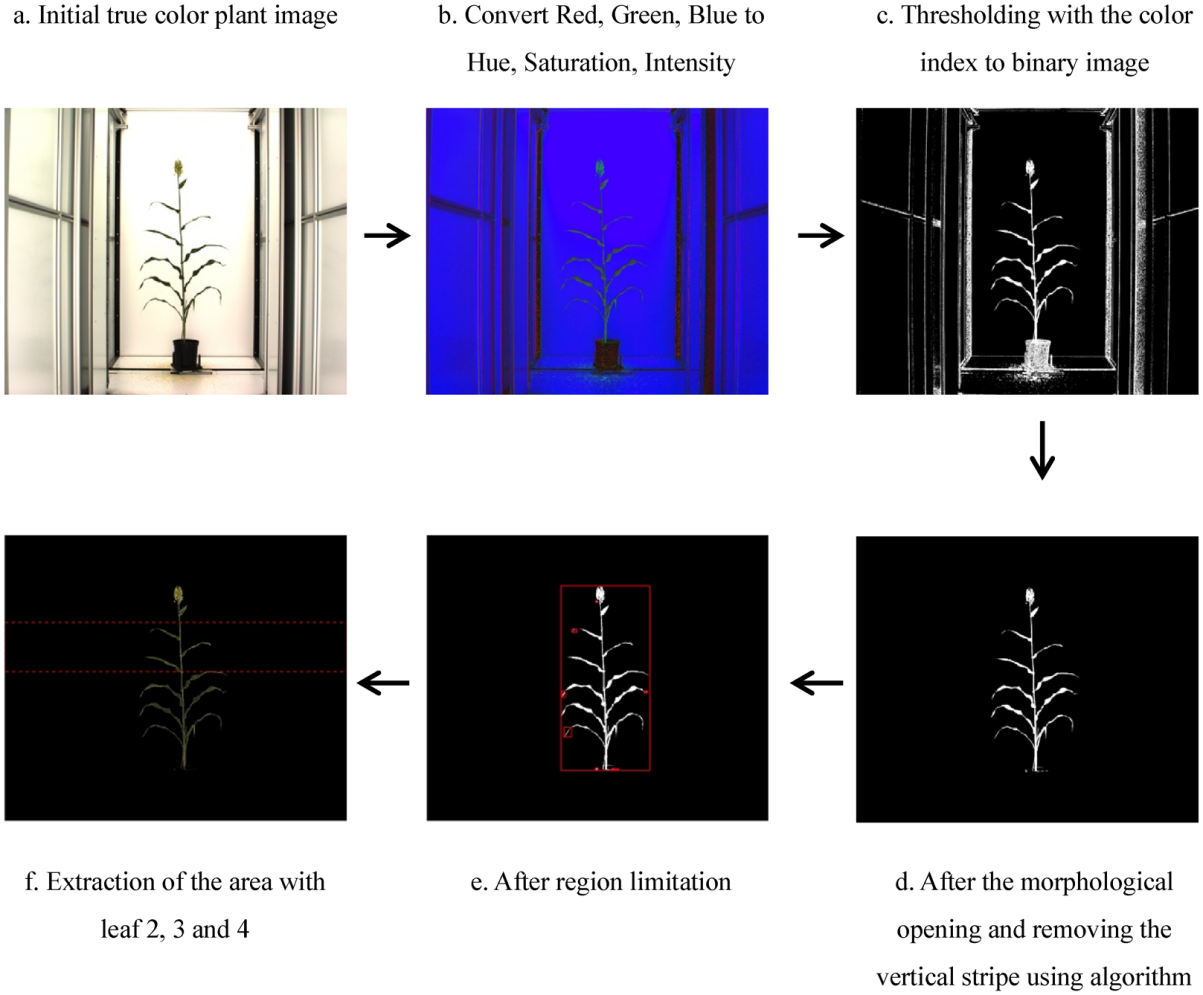
The sequential steps in segmentation of plant pixels from the background

Ten 2454×2056 resolution RGB images were taken of every plant: ten side view images from every 36 degrees at a horizontal rotation. In order to compare the estimation between chlorophyll content with color features, image processing technique was used and the color components of red (R), green (G) and blue (B) in RGB space and hue (H), saturation (S) and intensity (I) in HSI space were determined. During the color analysis, the HSI space was calculated by using the RGB space to increase the contrast between plant region and background region. Color spaces RGB and HSI can be transformed from one to another easily as illustrated in Eqs. (1)–(3). A schematic diagram of the image processing procedure is shown in Fig. 8.

1 $$\mathrm{H}=\mathrm{arccos}\left\{\frac{\left[\left(R-G\right)+(R-B)\right]/2}{{\left[{(R-G)}^{2}+(R-B)(G-B)\right]}^{1/2}}\right\}$$

2 $$\mathrm{S}=1-\frac{3}{(R+G+B)}\left[\mathrm{min}(R,G,B)\right]$$

3 $$\mathrm{I}=\frac{1}{3}(R+G+B)$$

Segmentation of these images was done by calculating a color index for each pixel and then using a threshold to derive a segmented image. The color index 3*S/(H + S + I) (where H, S, and I denote the hue, saturation and intensity components) was found to be effective in transforming HSI images to a single band images, because this index emphasized the saturation component in HSI pixels, and minimized the effect of non-consistent illumination among different images. A universal threshold of 0.75 was used to segment plant pixels from background.

The resulting image is a binary image, using white and black to distinguish the plant and background regions, respectively. But the binary image was found to contain noise in the form of isolated noise as well as vertical stripes near the edge of the image. Since the frames of the chamber were located in a fixed position for all images, the stripes were eliminated by using the reference image. Then morphological opening operation was applied to remove the small objects that contains fewer than 200 pixels from the binary image.

One important step in image processing is to determine the region in the images corresponding to Leaves 2, 3 and 4 of the plants. For this purpose, 300 RGB images were randomly drawn from the 3000 images (300 plants × 10 side-view RGB images for each plant). It was found that, among the selected 300 images, over 95% of the plants had their second leaf’s position below 5% of the plant height (measuring from the top). Similarly, over 95% of the plants had their fourth leaf’s position above 35% of the plant height. Therefore, the two thresholds, 5% and 35% in terms of the plant height, were used to define the region in which Leaves 2, 3 and 4 resided. We then acquired the extraction of the area with leaves 2, 3 and 4 to calculate the H, S, and I values, and used the same region to acquire the R, G, and B values from the original RGB image. Specific leaf image of hue (H) component was calculated as the total Hue value of image divided into the pixel number. Specific leaf image of saturation (S), intensity (I), red (R), green (G) and blue (B) component are in the same way.

**Fig. 9:**
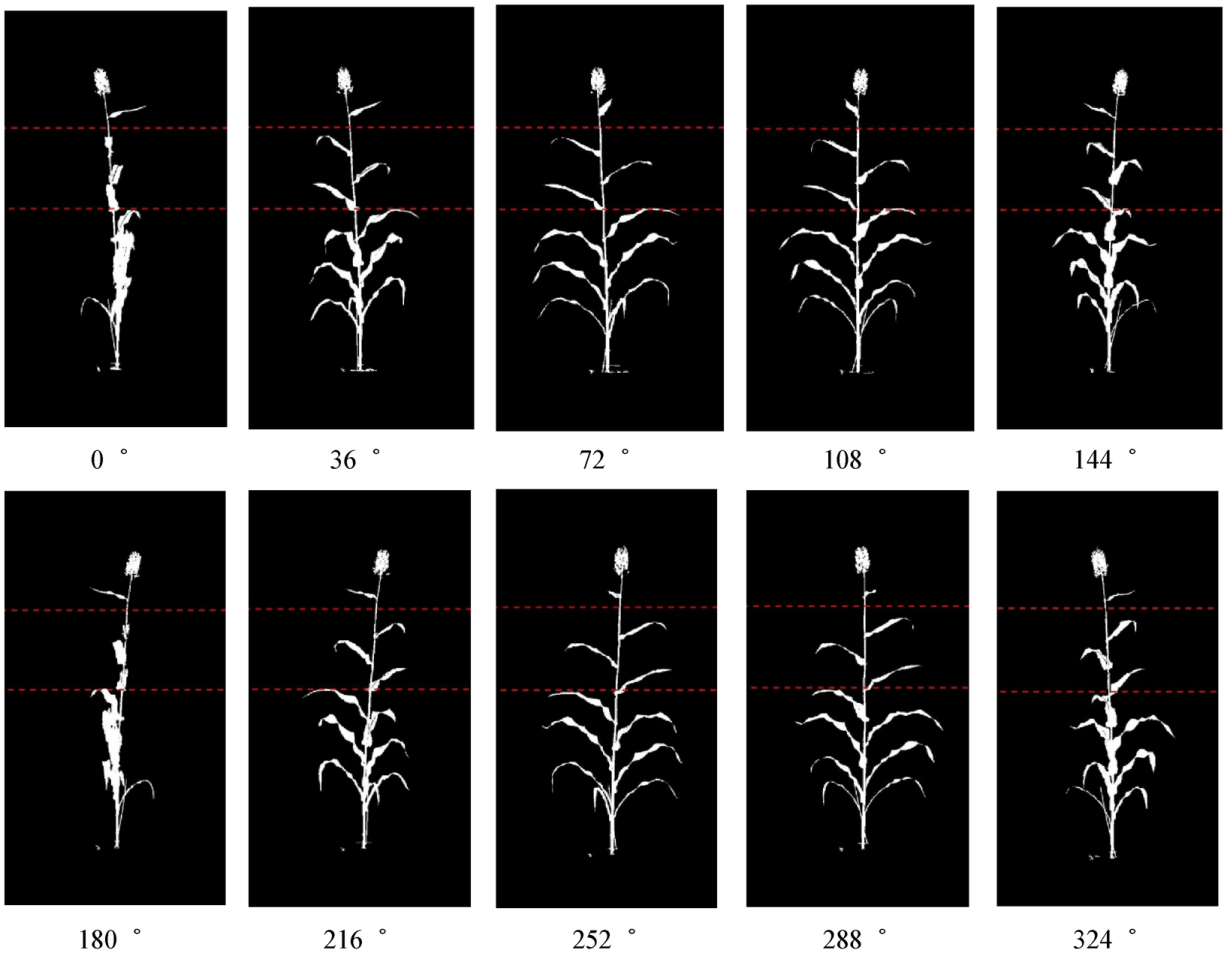
Ten different binary images converted from RGB from 0 to 360 degree

The total pixel count of the plant from ten side views were then averaged as plant Projected Area (PA, or equivalently, pixel count). The number of pixels inside the plant region was counted in each of the ten side views, and then averaged to give the projected shoot area. This is not the actual above-ground surface area but the average of the areas of the image projected in ten planes. There are many cases when a mature plant’s leaves are overlapping, appearing behind one another in side view images [17]. Figure 9 shows ten different binary images converted from RGB from 0 to 360 degree. The ten orthogonal views (ten side views from 36 rotational difference) provides a means of correction of plant area for those overlapping leaves, corrects for hidden areas in the other views and gives a robust representation of plant area overall.

#### Hyperspectral imaging acquisition

**Fig. 10:**
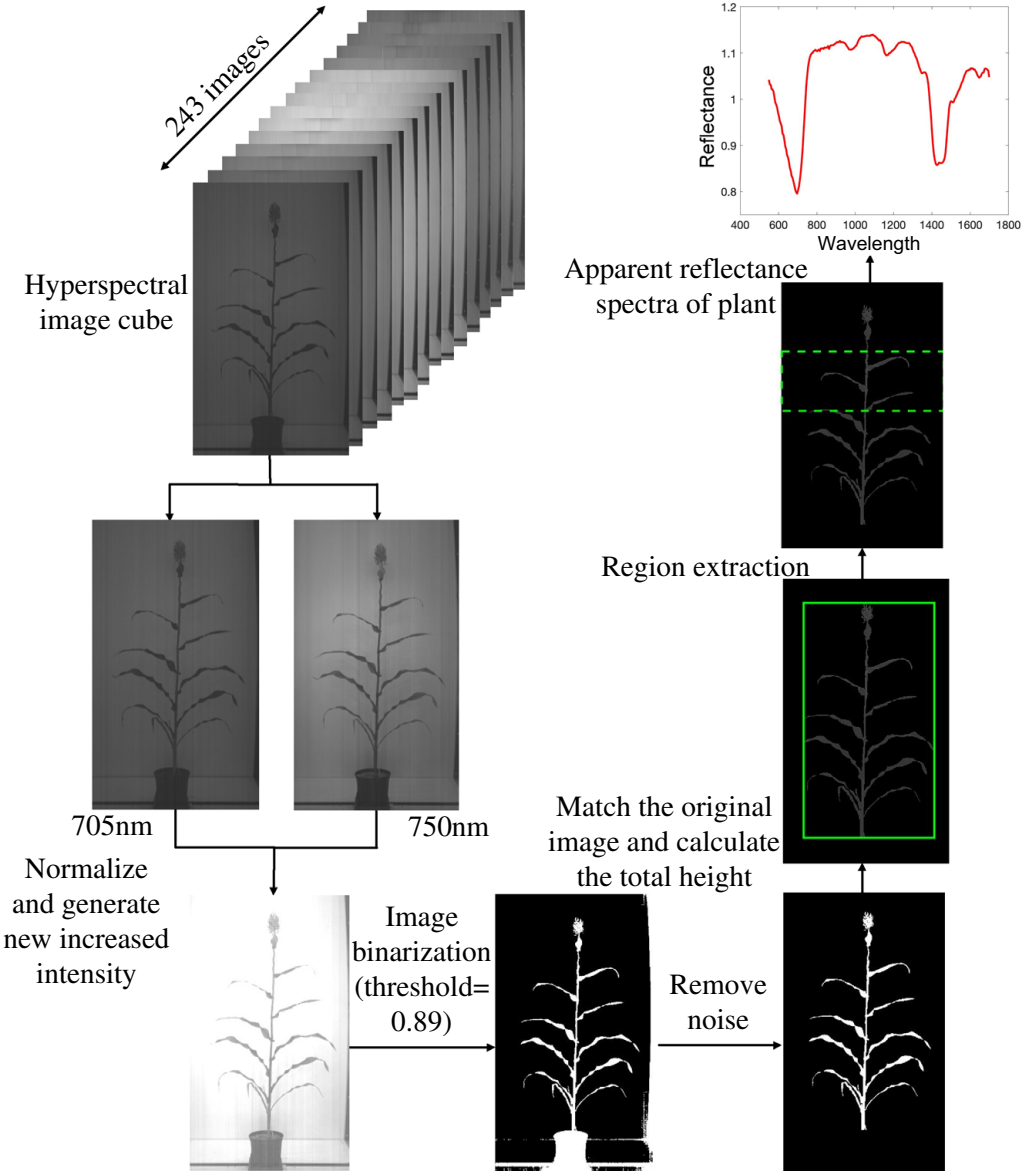
Flowchart showing the steps in hyperspectral image analysis to obtain apparent reflectance spectra

The hyperspectral image chamber consisted of a total of 243 image bands, with a spectral sampling resolution of 5 nm per band. The raw image was captured in a BIL (Band Interleaved by Line) format to acquire and store the original hyperspectral data. Then the individual spectral bands were then extracted from the BIL file using a custom MATLAB function to build 243 hyperspectral images [38]. The plant image cubes were individually processed to extract the spectrum of pixel intensities. The segmentation of plant pixels in the hyperspectral images was achieved by making use of the rapid increase in reflectance of vegetation [36]. The imagery was calibrated to reflectance [22]. The following procedures were used to process the hyperspectral images. Firstly, the segmentation process was accomplished. Then, pixels belonging to the plant region were selected for further processing. In order to separate the plant pixels from the non-plant pixels in the hyperspectral images, we used the two image bands with the following characteristics, one image band with the highest contrast between background and plant (705 nm) and the other image band with the lowest contrast (750 nm). Intensity of images at band 35 (705 nm) and band 44 (750 nm) were used to normalize and generate new increased intensity; and then we got a function of the sum of those new intensities. Second, the new intensities were applied to separate the plant pixels well from the non-plant pixels in the hyperspectral images. A global threshold of 0.89 was used to get a binary mask from this image, where the higher values belonged to the plant pixels. This binary mask was then used for segmentation of all image bands in the hyperspectral cube. After removal of noise, the binary image was matched to the original image, and specific leaf image of hyperspectral image was calculated as the total hyperspectral value of image divided into the pixel number. The complete hyperspectral image processing procedure was shown in Fig. 10.

The identification of plant stresses using hyperspectral imaging technology traditionally has been based upon changes in individual band intensities or changes in simple band ratios. In order to maximize the information reflected by the vegetation and minimize the impact of external factors, various hyperspectral indices were put forward and can detect subtle differences in physiological conditions and environmental stresses by their unique fine spectral characteristics, so these spectral indices have a broad application prospect in the inversion of chlorophyll content. The noninvasive optical methods all provided reliable estimates of relative leaf chlorophyll content. Empirical models to predict chlorophyll content are largely based on reflectance regions where the absorption is saturated at higher chlorophyll. Indices formulated with 705 nm and 750 nm bands would have higher accuracy in estimating chlorophyll content [16]. Reflectance index (chlorophyll content NDVI  =  $\left({R}_{750}-{R}_{705}\right)/\left({R}_{750}+{R}_{705}\right)$) were commonly used in the literature [38, 41]. Hyperspectral index can be divided into the following types: normalized spectral index, ratio spectral index, and multi-band spectral index. Algorithms such as the ratio vegetation index (RVI), the normalized difference vegetation index (NDVI), modified chlorophyll absorption ratio index (MCARI) have been used to measure canopy cover and chlorophyll content of plants. These ratios and algorithms are positively correlated to total chlorophyll levels in plants and each of them represents normalized spectral index, ratio spectral index, and multi-band spectral index. The most known and widely used vegetation index is NDVI. In addition, improved indices such as RVI and MCARI have been developed in order to combine the advantage of different vegetation index and improve the linearity relationship with vegetation biophysical variables. Three indices NDVI [750,705], RVI [750,705], and MCARI [750,705 and 550] [27, 30, 47] were tested in this study with the following formulate:

4 $$\mathrm{NDVI}=\frac{{R}_{750}-{R}_{705}}{{R}_{750}+{R}_{705}}$$

5 $$\mathrm{RVI}=\frac{{R}_{750}}{{R}_{705}}$$

6 $${\text{MCARI}} = \left( {\left( {R_{750} - R_{705} } \right) - 0.2*\left( {R_{750} - R_{550} } \right)} \right)* \, \left( {R_{750} /R_{705} } \right)$$

#### Fluorescence imaging acquisition

Fluorescence imaging captures the image that the red band is mainly emitted from photosystem. With this system, various fluorescence signals could be obtained. For each region of interest, the fluorescence parameter values of all pixels within the area were averaged. Many different fluorescence parameters were used to characterize the various aspects of photosynthetic performance comprehensively.

**Fig. 11:**
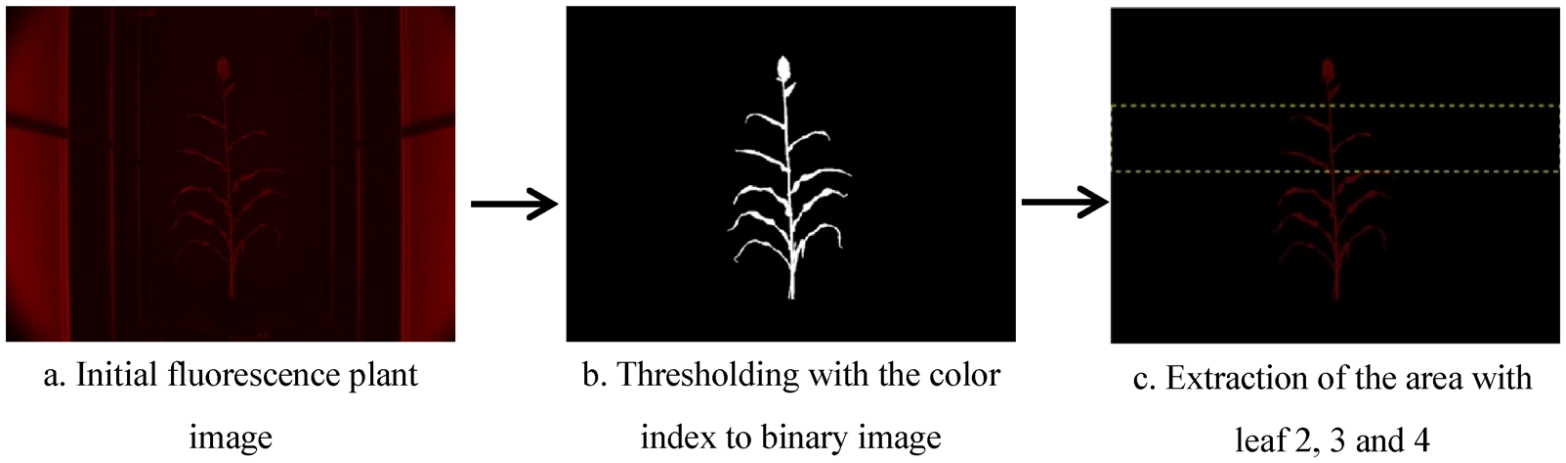
Process flow of image processing steps used in the extraction of plant’s projected chlorophyll content from the fluorescence images

Once chlorophyll fluorescence images were obtained, chlorophyll content of the entire plant was analyzed. For sorghum, the area of the entire plant was estimated using region of interest pixel numbers, and was used to monitor plant growth. A threshold value of 0.13 was determined to effectively segment plant pixels from the background. The fluorescence image of the plant from different degree side views were then averaged as plant projected area. Fifty images were randomly sampled from the fluorescence image dataset. For each image, 10 different pixel locations were randomly selected, with 5 locations indicating the non-plant pixels and 5 positions indicating the plant pixels. As a result, 250 positions for background and 250 positions for plants were obtained. The intensity for each locations was derived by converting the fluorescence intensity to a grayscale intensity in the fluorescence image. The critical threshold was then determined by choosing the intensity which was above the background upper limit and below the plant lower limit. A schematic diagram of the fluorescence image processing procedure was shown in Fig. 11. Specific leaf image of fluorescence image was the ratio of the total fluorescence value of image divided and the pixel number.

#### Data analysis

From the visible image, primary colors red (R), green (G) and blue (B), was recorded. Spectral parameters such as hue (H), saturation (S) and luminosity (L) were estimated from RGB values. Specific leaf image was calculated by dividing the total component of image by total pixel number. Hyperspectral and fluorescence image was processed in the same way. Specific leaf weight (SLW) is the oven-dry mass, divided by its one-sided area of fresh leaf (unit, g cm^−2^).

In this analysis, DAS (days after sowing) is measured from the date of planting. The visual appearances were confirmed by DAS, because DAS represent the growth stage development. Previous research reported that the first period occurring in seeding stage matched with chlorophyll content’s slow rise. The second critical period occurred in active jointing-booting growth stage and matched with chlorophyll content’s fast increase. The third period is filling to maturing stage matched with chlorophyll content’s slow decrease [2, 9]. DAS also showed a significant (p < 0.01) positive correlation with the macronutrient content [50]. Therefore, chlorophyll content could be written as a linear form of DAS and high-throughput image.

For each type of imaging, the 300 plants were split into two groups: 240 plants (80%) for model calibration and the other 60 (20%) for independent model validation. The split was done such that the different sorghum genotypes and the treatment levels for both water and nutrient were presented in the calibration and validation set. The following statistic was calculated using software R for model evaluation: Coefficient of Determination (R^2^) between for MC 100-measured and model-predicted chlorophyll content values. Akaike Information Criterion (AIC) is asymptotically equivalent to cross-validation and works to balance the trade-offs between the complexity of a given model and its goodness of fit. AIC is used to select the regression models that balance the size of the model and the predictive power. When choosing the best model from a set of alternative models, the smallest AIC is criterion.

To comprehensively include all the variables acquired form visible, hyperspectral and fluorescence images, partial least squared regression (PLSR) was used to model the sorghum plant chlorophyll content from image data. PLSR is constructed to illustrate the vegetation indexes' effect from different imaging techniques on the chlorophyll content. The variables, including hue, saturation, intensity, R, G, B, fluorescence, NDVI, RVI, MCARI. To find out the model with the best performance, fivefold cross-validation is applied to the training dataset, and therefore, the model with minimum mean square error is preferred. The following statistics were evaluated for model performance: *R*^*2*^, root mean squared error (RMSE, Eq. 7), and ratio of performance to deviation (RPD, Eq. 8) between the lab-measured and model-estimated plant chlorophyll content.

7 $$RMSE=\sqrt{\frac{1}{N}\times \sum_{i}{(\widehat{{y}_{i}}-{y}_{i})}^{2}}$$

8 $$RPD=\frac{SD(y)}{RMSE}$$

N is the number of plants in the calibration or validation set (300); *y*_*i*_ and $\widehat{{y}_{i}}$ are the lab-measured and model-predicted values, respectively; SD and mean are the standard deviation and mean of the lab-measured values.

## Data Availability

The datasets generated and analyzed during the current study are available from the corresponding author on reasonable request.

## Abbreviations

RGB: Red, green, blue
HSI: Hue, saturation, intensity
SLW: Specific leaf weight
DAS: Day after sowing
PLSR: Partial least squares regression
Chl: Chlorophyll content
NDVI: Normalized difference vegetation index
RVI: Ratio vegetation index
MCARI: Modified chlorophyll absorption ratio index
R^2^Coeffi: Cient of determination
RMSE: Root mean squared error
RPD: Ratio of performance to deviation
AIC: Akaike information criterion
